# A scientific approach to optimal treatment of cruciate ligament injuries

**DOI:** 10.3109/17453674.2011.588864

**Published:** 2011-07-08

**Authors:** M R Krogsgaard, J Brodersen, J Comins

**Affiliations:** Department of sportstraumatology and arthroscopy, Copenhagen University Hospital, Bispebjerg Copenhagen, Denmark and Department of general practice Faculty of health science Copenhagen University, Denmark

*Sir*—Which is the most effective treatment strategy for injuries to the anterior cruciate ligament (ACL) of the knee? This is the topic of an editorial in Acta Othopaedica in which Aspenberg (2010) reflects on a study by Frobell and colleagues (2010). The editorial headlines that ACL injuries are surgically over-treated. We think that this cannot be concluded, as there are severe problems with the measurement properties of the score systems that are used by Frobell et al.

Frobell et al. found no significant difference between ACL deficient patients randomized to a rehabilitation regimen with early ACL reconstruction and patients randomized to rehabilitation with delayed ACL reconstruction “when needed”. The outcome score was a modified version (KOOS-4) of the Knee injury and Osteoarthritis Outcome Score (KOOS).

We have shown that only 2 of the 5 subscales in the original version of KOOS fulfill the criteria of a unidimensional measurement scale when applied to ACL reconstructed patients (Comins et al. 2008). KOOS-4 differs from KOOS in that the domain to assess daily function (ADL) has been removed, and consists of 25 items distributed across 4 domains. The scores from each domain are added and divided by 4 to equally weight them. This summed and weighted total score is compared across groups (Frobell et al. 2010). But summing the scores from the separate subscales in KOOS is not justifiable (Comins et al. 2008). Among other problems we found ceiling effects in many items, and we concluded that KOOS is insufficient as a tool to evaluate function in ACL reconstructed patients 20 weeks after operation. There is no evidence to support the use of KOOS-4 to measure and compare outcome scores for these patients.

Tegner Activity Score and SF-36, which were also used in the study of Frobell et al. may have the same type of problems as KOOS regarding measurement properties (Hobart et al. 2002, Baron et al. 2006, Hagell et al. 2008).

In-depth interviews of 22 of the patients randomized in Frobells study to rehabilitation and optional reconstruction showed that many had joined the study to bypass the waiting list for surgery (Thorstensson et al. 2009). Patients who had different access to surgical treatment and who wanted early reconstruction would most likely not participate in this study. Of the patients who declined participation in the randomised study, the most common reason was unwillingness to undergo surgery (Frobell et al. 2007). Patients with the most and least severe injuries might be included less frequently in the study compared to patients with moderate injuries and this could skew results.

Whichever treatment is performed, an ACL lesion has significant consequences. In a 20- year follow-up of 19 Olympic athletes who sustained an ACL injury between 1963 and 65 and were treated non-operatively due to a protocol in the former East Germany, 18 had meniscal resection performed and 13 had grade IV chondral lesions at arthroscopy. By the end of 2000, 10 of the athletes had a knee replacement (Nebelung and Wuschech 2005). In 100 patients with an acute ACL injury, 22 were reconstructed, and of 67 patients followed for 15 years, 21% had a poor or fair result, and generally there was a significant decrease in Tegner and Lysholm scores between 3 and 15 years post-injury (Kostogiannis et al. 2007). Despite ACL-reconstruction, more than 50% have not returned to preinjury activity levels after 12 months (Ardern et al. 2010). The effect of non-operative treatment after ACL injury has been reported in several studies. Dependent on the selection of patients, up to 2/3 are treated later with an ACL-reconstruction (Strehl and Eggli 2007). Early ACL reconstruction is followed by better functional results compared to delayed reconstruction (Ahlén and Lidén 2010). Operated patients have better subjective and objective stability and a higher chance of returning to preinjury level of sports (Hinterwimmer et al. 2003), however a Cochrane review (Linko et al. 2005) concluded, that there is not enough evidence to recommend operative or non-operative treatment.

It is not clear what non-operative treatment should consist of. After ACL-reconstruction a home-based program is as good as a treatment program in a physical therapy clinic to restore knee function (Grant et al. 2010). In a randomized study between self-monitored training in a clinic and supervised training by a physical therapist after ACL injury, a large number of patients were transferred to the supervised group before follow-up, so the study results are difficult to interpret (Zätterström et al. 2000). No study has compared non-treatment with physical therapy. The non-operative treatment strategy of rehabilitation first and ACL reconstruction when deemed necessary is time consuming for the patients who choose reconstruction, as they must participate in rehabilitation twice (Thorstensson et al. 2009).

In patients with ACL rupture meniscal injury is the most important risk factor for osteoarthritis (Öiestad et al. 2009). Stabilization of the knee through ACL reconstruction could reduce the risk of meniscus injury.

We believe that the literature, including Frobell's recent study, does not provide a basis to conclude that there is surgical overtreatment of cruciate ligament injuries. At present there is no documented best treatment of acute ACL injuries.
