# Supplementary material for: A scientific approach to optimal treatment of cruciate ligament injuries
Source: Acta Orthop. 2011 Jul 8;82(3):389–92. doi: 10.3109/17453674.2011.588864 (PMC3235324; doi:10.3109/17453674.2011.588864)
Supplement: Related texts

# Letter: A scientific approach to optimal treatment of cruciate ligament injuries

**Published:** 2011-07-08

**Authors:** Per Aspenberg

**Affiliations:** Orthopedics, AIR/IKE, Faculty of Health Science, Linköping University, Linköping, Sweden

*Sir*—I thank Dr. Krogsgaard and colleagues for their interest in my Editorial on anterior cruciate ligament (ACL) injuries.

I have asked Drs Lohmander and Frobell to comment on the critique of their methods. I note that both Krogsgaard et al. and Lohmander et al. agree with the Cochrane review conclusion that there is no documented best treatment of acute ACL injuries. Considering this lack of evidence, I believe it justified to state that surgery in many cases might represent overtreatment.

# Letter: A scientific approach to optimal treatment of cruciate ligament injuries

**Published:** 2011-07-08

**Authors:** L Stefan Lohmander, Richard B Frobell, Ewa M Roos

**Affiliations:** Department of Orthopedics, Clinical Sciences Lund, Lund University, Sweden, and Research Unit for Musculoskeletal Function and Physiotherapy and Department of Orthopaedics and Traumatology, University of Southern Denmark, Denmark; Department of Orthopedics, Clinical Sciences Lund, Lund University, Sweden; Research Unit for Musculoskeletal Function and Physiotherapy, Institute of Sports Science and Clinical Biomechanics, University of Southern Denmark, Denmark

*Sir*—We thank Dr. Krogsgaard and colleagues for their continued interest in our study on anterior cruciate ligament (ACL) injuries.

At 2 years of follow-up our randomized controlled trial showed no advantage of early ACL reconstructive surgery with structured rehabilitation over that of structured rehabilitation with optional reconstruction ‘as needed’, as monitored by the pre-specified primary outcome: change from baseline to 2 years in the average score of 4 of the 5 KOOS subscales, covering pain, symptoms, difficulty in sports and recreational activities, and knee-related quality of life (KOOS-4, equal weight for all subscales) (Frobell et al. 2010).

Further, we found no difference at 2 years for any of the pre-specified secondary outcomes: each of the 5 individual KOOS subscales (the fifth scale being activities of daily living), the scores on the SF-36 physical and mental components, the Tegner activity scale, the area under the curve for the development of absolute KOOS4 scores from baseline to 2 years, and the percentage of subjects with a KOOS quality-of-life score below 44 (a pre-specified cutoff value consistent with a report of more than moderately decreased knee-related quality of life) between 6 months and 2 years. Neither was there any difference between the groups in the number of meniscal surgeries during the 2-year follow-up. As expected, subjects assigned to rehabilitation plus early ACL reconstruction had greater knee stability at 2 years shown by the exploratory outcomes Lachman test, pivot shift test, and KT1000 arthrometry. Our post hoc as-treated analysis identified no significant differences in self-reported outcomes at 2 years among the subjects treated with rehabilitation plus early ACL reconstruction, those treated with rehabilitation plus delayed ACL reconstruction, and those treated with rehabilitation alone.

Krogsgaard and colleagues discuss several different issues in their letter. Firstly, they question the validity of the KOOS questionnaire, the SF-36 and the Tegner activity scale to measure and compare outcomes of patients with ACL injuries.

As noted in our previous response to this question (Comins et al. 2010), we agree that the KOOS subscale for “function in daily living” has poor content validity in patients with acute ACL injury, and thus did not include it in our pre-specified primary outcome of this trial. We emphasize that the analyses of each of the individual KOOS subscales failed to show a difference between the study groups, consistent with the primary outcome. The finding of large ceiling effects of the KOOS subscales reported by Comins and colleagues is not consistent across populations and was not replicated in a recent validation study of ACL reconstructed subjects (Salavati et al. 2011). In contrast to the Tegner Activity Scale, the KOOS does not require adjustment for age and sex in amateur soccer players (Frobell et al. 2008), or for different cultures (Magnussen et al. 2010). Finally, usage of the KOOS in large scale databases such as the Scandinavian ACL registries (Granan et al. 2009) and in the US MOON database (Dunn et al. 2010) enables not only cross cultural comparisons but also provides reference data (Ageberg et al. 2010). Consequently, a recent meta-analysis recommends the KOOS for monitoring outcome following ACL surgery (Wang et al. 2010).

The SF-36 is a widely used generic instrument to assess health-related quality of life. We have previously shown SF-36 to be responsive in groups having orthopedic surgery interventions, including ACL reconstruction, but caution against use in individual patients due to a large proportion of individuals being affected by floor and ceiling effects and low individual sensitivity to change (Busija et al. 2008).

While the measurement properties of the Tegner activity scale may be less than perfect, it is widely used to report physical activity following knee injuries; a PubMed search identifies more than 100 studies relevant to this discussion.

Secondly, Krogsgaard and colleagues suggest the presence of bias in the patient screening and inclusion in our study. During the inclusion period, we screened all patients with acute rotational trauma to the knee. All patients fulfilling the predefined inclusion and exclusion criteria were examined by an experienced clinician for the presence of antero-posterior laxity (i.e. positive Lachman test) and an ACL tear visualized on MRI confirmed the diagnosis (Frobell et al. 2010). Out of 196 eligible patients thus identified, 55 declined to participate: 30 were not willing to risk undergoing ACL reconstruction, 10 not willing to undergo non-surgical treatment, and 15 unwilling to participate in the trial. As reported, the characteristics of those who declined participation (for any reason) were similar to those who accepted (Frobell et al. 2010). The overall KOOS results at 2 years were similar to those in other ACL reconstruction studies (Nau et al. 2002, Aglietti et al. 2004, Beynnon et al. 2005, Granan et al. 2008, Lind et al. 2009) and to KOOS-4 results 2 years after surgery for patients of similar age included in the Swedish National ACL Register (Ageberg et al. 2010, Ageberg E: personal communication). The median Tegner activity scores 2 years after ACL reconstruction in our study were also similar to those reported by other investigators (Eriksson et al. 2001, Ejerhed et al. 2003, Jansson et al. 2003, Laxdahl et al. 2005). These similarities in outcomes between our study and those reported by other investigators suggest that our findings are generalizable.

With regard to the risk of meniscus lesions and other knee re-injury in reconstructed and non-reconstructed knees, we refer to our previous response in Acta to the letter of Løken et al. (2011). Observational studies suffer from confounding by indication, and the only randomized controlled trial showed no difference at 2 years (Frobell et al. 2010). More evidence is needed to draw any conclusions on the benefit of one or the other treatment in this respect.

Consistent with our publication record, we fully agree with Krogsgaard and colleagues on the often serious long-term consequences of an injury to the ACL, in particular the development of osteoarthritis (Lohmander and Roos 1994, Roos et al. 1995a, b, Lohmander et al. 2004, 2007, von Porat et al. 2004, Neuman et al. 2008). We are pleased to note the increasing attention to this problem, as compared to the frequent use of short-term consequences of “return to play” or “pre-injury activity” level as outcomes in studies of ACL injury. However, there is insufficient evidence to show whether ACL reconstruction is associated with less or more post-injury osteoarthritis compared with non-surgical management (Lohmander et al. 2007, Meuffels et al. 2009).

We agree with the conclusion of the Cochrane systematic review of Linko et al. 2005): “There is insufficient evidence from randomised trials to determine whether surgery or conservative management was best for ACL injury in the 1980s, and no evidence to inform current practice. Good quality randomised trials are required to remedy this situation”. We have now published one such trial, showing no advantage at 2 years of the addition of ACL reconstruction to structured rehabilitation. More long-term trials are needed to confirm or refute these results in other populations. We continue to monitor our patients, and will report on longer-term outcomes.

## References

[CIT0001] Ageberg E, Forssblad M, Herbertsson P, Roos EM (2010). Sex differences in patient-reported outcomes after anterior cruciate ligament reconstruction: data from the Swedish knee ligament register. Am J Sports Med.

[CIT0002] Aglietti P, Giron F, Buzzi R, Biddau F, Sasso F (2004). Anterior cruciate ligament reconstruction: bone-patellar tendon-bone compared with double semitendinosus and gracilis tendon grafts: a prospective, randomized clinical trial. J Bone Joint Surg (Am).

[CIT0003] Ahlén M, Lidén M (2010). A comparison of the clinical outcome after anterior cruciate ligament reconstruction using a hamstring tendon autograft with special emphasis on the timing of the reconstruction. Knee Surg Sports Traumatol Arthrosc.

[CIT0004] Ardern CL, Webster KE, Taylor NF, Feller JA (2010). Return to the preinjury level of competitive sport after anterior cruciate ligament reconstruction surgery: Two-thirds of patients have not returned 12 months after surgery. Am J Sports Med.

[CIT0005] Aspenberg P (2010). Overtreatment of cruciate ligament injuries. Acta Orthop.

[CIT0006] Baron R, Elashaal A, Germon T, Hobart J (2006). Measuring outcomes in cervical spine surgery: think twice before using the SF-36. Spine.

[CIT0007] Beynnon BD, Uh BS, Johnson RJ, Abate JA, Nichols CE, Fleming BC, Poole AR, Roos H (2005). Rehabilitation after anterior cruciate ligament reconstruction: a prospective, randomized, double-blind comparison of programs administered over 2 different time intervals. Am J Sports Med.

[CIT0008] Busija L, Osborne RH, Nilsdotter AK, Buchbinder R, Roos EM (2008). Magnitude and meaningsfulness of change in SF-36 scores in four types of orthopaedic surgery. Health Qual Life Outcomes.

[CIT0009] Comins J, Brodersen J, Krogsgaard M, Beyer N (2008). Rasch analysis of the knee injury and osteoarthritis outcome score (KOOS): a statistical re-evaluation. Scand J Med Sci Sports.

[CIT0010] Comins J, Brodersen J, Krogsgaard M (2010). Treatment for acute anterior cruciate ligament tear. New Engl J Med.

[CIT0011] Dunn WR, Spindler KP, Amendola A, Andrish JT, Kaeding CC, Marx RG, McCarty EC, Parker RD, Harrell F, An AQ, Wright RW, Brophy RH, Matava MJ, Flanigan DC, Huston LJ, Jones MH, Wolcott ML, Vidal AF, Wolf BR (2010). MOON ACL Investigation. Which preoperative factors, including bone bruise, are associated with knee pain/symptoms at index anterior cruciate ligament reconstruction (ACLR)? A Multicenter Orthopaedic Outcomes Network (MOON) ACLR Cohort Study. Am J Sports Med.

[CIT0012] Ejerhed L, Kartus J, Sernert N, Kohler K, Karlsson J (2003). Patellar tendon or semitendinosus tendon autografts for anterior cruciate ligament reconstruction? A prospective randomized study with a two-year follow-up. Am J Sports Med.

[CIT0013] Eriksson K, Anderberg P, Hamberg P, Löfgren AC, Bredenberg M, Westman I, Wredmark T (2001). A comparison of quadruple semitendinosus and patellar tendon grafts in reconstruction of the anterior Cruciate ligament. J Bone Joint Surg (Br).

[CIT0014] Frobell RB, Lohmander LS, Roos EM (2007). The challenge of recruiting patients with anterior cruciate ligament injury of the knee into a randomized clinical trial comparing surgical and non-surgical treatment. Contemp Clin Trials.

[CIT0015] Frobell RB, Svensson E, Gottrick M, Roos EM (2008). Self-reported activity level and knee function in amateur soccer players: the influence of age, gender, history of knee injury and level of competition. Knee Surg Sports Traumatol Arthrosc.

[CIT0016] Frobell RB, Roos EM, Roos HP, Ranstam J, Lohmander LS (2010). A randomized trial of treatment for acute anterior cruciate ligament tear. New Engl J Med.

[CIT0017] Granan LP, Bahr R, Steindal K, Furnes O, Engebretsen L (2008). Development of a national cruciate ligament surgery registry: the Norwegian National Knee Ligament Registry. Am J Sports Med.

[CIT0018] Granan LP, Forssblad M, Lind M, Engebretsen L (2009). The Scandinavian ACL registries 2004-2007: baseline epidemiology. Acta Orthop.

[CIT0019] Grant JA, Mohtadi NG (2010). Two- to 4-year follow-up to comparison of home versus physical therapy-supervised rehabilitation programs after anterior cruciate ligament reconstruction. Am J Sports Med.

[CIT0020] Hagell P, Tornqvist AL, Hobart J (2008). Testing the SF-36 in Parkinson's disease. Implications for reporting rating scale data. J Neurol.

[CIT0021] Hinterwimmer S, Engelschalk M, Sauerland S, Eitel F, Mutschler W (2003). Operative or conservative treatment of anterior cruciate ligament rupture: a systematic review of the literature. Unfallchirurg.

[CIT0022] Hobart JC, Williams LS, Moran K, Thompson AJ (2002). Quality of life measurement after stroke: uses and abuses of the SF-36. Stroke.

[CIT0023] Jansson KA, Linko E, Sandelin J, Harilainen A (2003). A prospective randomized study of patellar versus hamstring tendon autografts for anterior cruciate ligament reconstruction. Am J Sports Med.

[CIT0024] Kostogiannis I, Ageberg E, Neuman P, Dahlberg L, Fridén T, Roos H (2007). Activity level and subjective knee function 15 years after anterior cruciate ligament injury: a prospective, longitudinal study of nonreconstructed patients. Am J Sports Med.

[CIT0025] Laxdal G, Kartus J, Hansson L, Heidvall M, Ejerhed L, Karlsson J (2005). A prospective randomized comparison of bone-patellar tendon-bone and hamstring grafts for anterior cruciate ligament reconstruction. Arthroscopy.

[CIT0026] Lind M, Menhert F, Pedersen AB (2009). The first results from the Danish ACL reconstruction registry: epidemiologic and 2 year follow-up results from 5,818 knee ligament reconstructions. Knee Surg Sports Traumatol Arthrosc.

[CIT0027] Linko E, Harilainen A, Malmivaara A, Seitsalo S (2005). Surgical versus conservative interventions for anterior cruciate ligament ruptures in adults. Cochrane Database Syst Rev.

[CIT0028] Lohmander LS, Roos H (1994). Knee ligament injury, surgery and osteoarthrosis - Truth or consequences?. Acta Orthop Scand.

[CIT0029] Lohmander LS, Östenberg A, Englund M, Roos H (2004). High prevalence of knee osteoarthritis, pain, and functional limitations in female soccer players twelve years after anterior cruciate ligament injury. Arthritis Rheum.

[CIT0030] Lohmander LS, Englund M, Dahl LL, Roos EM (2007). The Long-term Consequence of Anterior Cruciate Ligament and Meniscus Injuries: Osteoarthritis. Am J Sports Med.

[CIT0031] Løken S, Årøen A, Engebretsen L (2011). Overtreatment of cruciate ligament injuries. Acta Orthop.

[CIT0032] Magnussen RA, Granan LP, Dunn WR, Amendola A, Andrish JT, Brophy R, Carey JL, Flanigan D, Huston LJ, Jones M, Kaeding CC, McCarty EC, Marx RG, Matava MJ, Parker RD, Vidal A, Wolcott M, Wolf BR, Wright RW, Spindler KP, Engebretsen L (2010). Cross-cultural comparison of patients undergoing ACL reconstruction in the United States and Norway. Knee Surg Sports Traumatol Arthrosc.

[CIT0033] Meuffels DE, Favejee MM, Vissers MM, Heijboer MP, Reijman M, Verhaar J AN (2009). Ten year follow-up study comparing conservative versus operative treatment of anterior cruciate ligament ruptures. A matched-pair analysis of high level athletes. Br J Sports Med.

[CIT0034] Nau T, Lavoie P, Duval N (2002). A new generation of artificial ligaments in reconstruction of the anterior cruciate ligament: two-year follow-up of a randomised trial. J Bone Joint Surg (Br).

[CIT0035] Nebelung W, Wuschech H (2005). Thirty-five years of follow-up of anterior cruciate ligament- deficient knees in high-level athletes. Arthroscopy.

[CIT0036] Neuman P, Englund M, Kostogiannis I, Fridén T, Roos H, Dahlberg LE (2008). Prevalence of tibiofemoral osteoarthritis 15 years after nonoperative treatment of anterior cruciate ligament injury: a prospective cohort study. Am J Sports Med.

[CIT0037] Roos H, Adalberth T, Dahlberg L, Lohmander LS (1995a). Osteoarthrosis of the knee after injury to the anterior cruciate ligament or meniscus - the influence of time and age. Osteoarthritis Cartilage.

[CIT0038] Roos H, Ornell M, Gärdsell P, Lohmander LS, Lindstrand A (1995b). Soccer after anterior cruciate ligament injury - an incompatible combination? A national survey of incidence and risk factors and a 7-year follow-up. Acta Orthop Scand.

[CIT0039] Salavati M, Akhbari B, Mohammadi F, Mazaheri M, Khorrami M (2011). Knee injury and Osteoarthritis Outcome Score (KOOS); reliability and validity in competitive athletes after anterior cruciate ligament reconstruction. Osteoarthritis Cartilage.

[CIT0040] Strehl A, Eggli S (2007). The value of conservative treatment in ruptures of the anterior cruciate ligament (ACL). J Trauma.

[CIT0041] Thorstensson CA, Lohmander LS, Frobell RB, Roos EM, Gooberman-Hill R (2009). Choosing surgery: patients' preferences within a trial of treatments fore anterior cruciate ligament injury. A qualitative study. BMC Musculoskelet Disord.

[CIT0042] von Porat A, Roos EM, Roos H (2004). High prevalence of osteoarthritis 14 years after an anterior cruciate ligament tear in male soccer players: a study of radiographic and patient relevant outcomes. Ann Rheum Dis.

[CIT0043] Wang D, Jones MH, Khair MM, Miniaci A (2010). Patient-reported outcome measures for the knee. J Knee Surg.

[CIT0044] Zätterström R, Fridén T, Lindstrand A, Moritz U (2000). Rehabilitation following acute anterior cruciate ligament injuries – a 12-month follow-up of a randomized clinical trial. Scand J Med Sci Sports.

[CIT0045] Öiestad BE, Engebretsen L, Storheim K, Risberg MA (2009). Knee osteoarthritis after anterior cruciate ligament injury: a systematic review. Am J Sports Med.
